# The Relationship Between Social Media Use and Health-Seeking Behaviors and Perceptions of Health Among Surgical Patients: A Descriptive and Cross-Sectional Study

**DOI:** 10.3390/healthcare14152283

**Published:** 2026-07-27

**Authors:** Burcak Sahin Koze, Yesire Alan, Bilgenur Eker

**Affiliations:** 1 Department of Surgical Nursing, Faculty of Nursing, Ege University, Bornova-Izmir 35100, Türkiye; 2 Palliative Care Center, Beylikdüzü State Hospital, Istanbul Provincial Health Directorate, Istanbul 34500, Türkiye; yesirealan@gmail.com; 3 Department of Ophthalmology, Izmir City Hospital, Izmir Provincial Health Directorate, Izmir 35540, Türkiye; bilgeeker202@gmail.com

**Keywords:** health literacy, health seeking behavior, internet, social media

## Abstract

**Background/Objectives**: The primary goal of this research was to investigate the relationship between the utilization of social media platforms, its influences on health status perceptions and health information-seeking tendencies of individuals who underwent surgical procedures. **Methods:** This study was designed and executed utilizing a descriptive and cross-sectional design. The research cohort comprised 376 surgical patients selected from operative clinics who satisfied the specified eligibility requirements for participation. Data collection was carried out through a structured Patient Demographic Questionnaire alongside the Health-Seeking Behavior and Health Perception Scales. For statistical evaluation of the gathered data, descriptive metrics, both parametric and non-parametric analytical techniques, and correlation tracking frameworks were employed. **Results:** According to the study analysis, participants under the age of 54 achieved significantly higher web-based health information-seeking scores (15.79 ± 5.50) than those in the older demographic group (14.10 ± 5.83; t = 2.886, *p* < 0.05). In terms of gender variations, female patients demonstrated more prominent scores in both digital and comprehensive health-seeking tasks compared to male counterparts (t = 2.316, t = 2.130; *p* < 0.05). Academic background elicited significant statistical fluctuations across all sub-dimensions (F = 5.655, F = 7.080, F = 7.690, F = 9.877; *p* < 0.001), revealing that individuals with only primary school education presented the lowest baseline values. Furthermore, individuals restricting their daily internet exposure to one hour or less demonstrated noticeably lower scores than peers maintaining an extended daily digital presence (F = 4.791, F = 4.232, F = 3.549; *p* < 0.05). **Conclusions:** This study found that women and individuals with higher levels of education exhibit more frequent online health-seeking behaviors, which were found to be more prominent among individuals who spend more time online and actively use social media platforms.

## 1. Introduction

Technological advancements have brought about profound changes in people’s daily lives, fundamentally altering how health-related information is accessed and interpreted. The rapid evolution of the information society has significantly affected the paradigm of health and illness management [1,2]. Driven by widespread internet and social media usage, individuals increasingly seek online information regarding their own medical conditions and symptoms. This shift has introduced both positive opportunities and critical challenges. While expanded internet access empowers individuals to take greater control over their healthcare decisions [2,3], the vast influx of unverified data on social platforms poses substantial risks [4]. Not every social media post is evidence-based, and because clinical symptoms and treatment decisions require individualized clinical assessment, relying on generalized online claims may expose surgical patients to misinformation and inappropriate health decisions [5].

A pivotal study by Can and colleagues [6] examining health-related websites determined that a significant portion of the analyzed platforms lacked evidence-based information, more than half excluded expert opinions, and the majority of those including expert views failed to cite credible references. This deficiency not only undermines the reliability of online health resources but also misleads individuals, occasionally driving them toward incorrect self-diagnoses and hazardous self-treatment practices that deteriorate their clinical status [2,7]. This issue becomes particularly acute in perioperative care. Patients scheduled for surgery frequently experience profound pre-operative anxiety due to uncertainty surrounding the impending procedure. To mitigate this psychological burden, they require structured, comprehensive information regarding the pre-, intra-, and post-operative periods [8]. When patients’ specific informational needs are adequately met, their anxiety levels decrease, thereby positively influencing the overall surgical recovery trajectory [9,10]. Consequently, online resources directly shape the health-seeking behaviors of surgical patients, heavily influencing how they comprehend their diagnosis, treatment options, and recovery phases [1,5,11].

Evaluating this phenomenon specifically within surgical candidates is of paramount clinical importance, as these patients represent a highly vulnerable population facing acute preoperative anxiety, invasive procedures, and a critical need for accurate perioperative instructions. Misinformation sourced from digital platforms can directly compromise informed consent, alter patient expectations, and jeopardize postoperative compliance. While recent global nursing literature confirms that social media can facilitate peer support, it concurrently emphasizes that the internet remains a double-edged sword [12,13,14,15,16,17]. A critical gap remains in understanding how these digital health-seeking tendencies directly interact with distinct health perceptions in patients undergoing surgical interventions. Accordingly, our study aimed to investigate the relationship between the utilization of the internet/social media and the health-seeking behaviors and health perceptions among surgical patients.

## 2. Materials and Methods

### 2.1. Type of Study and Research Questions

This study utilized a descriptive and cross-sectional research design. This specific design was selected as it is highly appropriate for simultaneously exploring and describing the baseline relationships and current patterns between patients’ health-seeking behaviors and their health perceptions at a single point in time, without manipulating variables or establishing definitive causal mechanisms.

The research questions addressed in this study are as follows:

- What are patients’ health-seeking behaviors?
- What are patients’ perceptions of health?
- Does a correlation exist between the way patients seek healthcare and how they perceive health?

### 2.2. Study Setting and Time

This study was conducted between December 2022 and June 2023 at a university hospital in western Türkiye.

### 2.3. Population and Sampling

The target population of this research comprised approximately 6000 inpatients receiving treatment across various operative departments—including General Surgery, Neurosurgery, Urology, Organ Transplantation, Cardiovascular Surgery, Thoracic Surgery, Orthopedics and Traumatology, Plastic, Reconstructive, and Aesthetic Surgery, Otolaryngology, Ophthalmology, and Obstetrics and Gynecology—at a university hospital situated in the western region of Turkey. To determine the minimum required sample size from this finite population, the sample size formula for a known population was utilized, assuming a 95% confidence level, and a 5% margin of error. Although the minimum representative sample size was calculated as 361, a convenience sampling method was employed to recruit participants to account for potential data loss. Ultimately, the final study sample was composed of 376 patients who successfully met all specified eligibility requirements and voluntarily agreed to participate, thereby exceeding the calculated minimum sample threshold. Patients were selected for enrollment based on meeting the following criteria: being at least 18 years old, having completed a surgical intervention within the facility, possessing literacy and fluency in the Turkish language, having operational familiarity with digital hardware (smartphones, computers, or tablets) combined with active network connectivity, and providing voluntary agreement for trial tracking.

### 2.4. Data Collection

To gather the research data, the investigators utilized a researcher-designed Individual Profile Form alongside two established scales selected from the literature: the Health-Seeking Behavior Scale and the Health Perception Scale. Data collection took place from December 2022 to June 2023 via in-person interviews with the participants. Prior to administration, patients were briefed on this study’s objectives, and their written informed consent was secured through a formal consent form. Filling out the assessment tools required roughly 10 min per patient.

Personal information form: The sociodemographic characteristics of patients who underwent surgery (such as age, gender, educational status, employment status, marital status, whether they have children, and income level), general health status (presence of chronic diseases, history of previous surgeries, current diagnosis, the clinic where they received treatment, their knowledge of the illness, sources of information about the illness, and the most helpful information source), and questions regarding internet and social media usage (devices used to access the internet, social media usage patterns, time spent online, history of conducting online research for any previous health issues, current online research regarding their illness, and trust in health-related information found online) for a total of 25 questions.

Health-Seeking Behavior Scale (HSBS): The evaluation of participants’ health-seeking tendencies was conducted using the tool designed by Kıraç [18], which contains 12 separate items spread across three primary dimensions. These structural dimensions are specified as “online health-seeking behavior,” “professional health-seeking,” and “traditional health-seeking behavior.” Regarding the internal consistency metrics of the instrument, the overall Cronbach’s alpha coefficient was determined to be 0.755, whereas the independent sub-dimension alpha values were recorded as 0.726, 0.720, and 0.736, respectively [18]. The instrument utilizes a traditional 5-point Likert-type scoring framework, where individual question weights range from 1 to 5.

Perception of Health Scale (PHS): To examine health perception levels, the scale formulated by Diamond et al. [19] was utilized. This assessment tool consists of 15 items grouped under four distinct conceptual subscales: locus of control, certainty, importance of health, and self-awareness. Respondents rate each statement using a 5-point Likert scale (spanning from “strongly agree = 5” to “strongly disagree = 1”), with adverse items being reverse-scored prior to calculation. The cumulative score obtainable from the complete instrument varies between 15 and 75, where elevated total outcomes signify a more optimized or positive health perception. The cultural adaptation, validity, and reliability assessments for the Turkish version of this instrument were executed by Kadıoğlu and Yıldız [20]; within the scope of the present study, the overall Cronbach’s alpha value for this scale was computed as 0.77.

**Figure 1:**
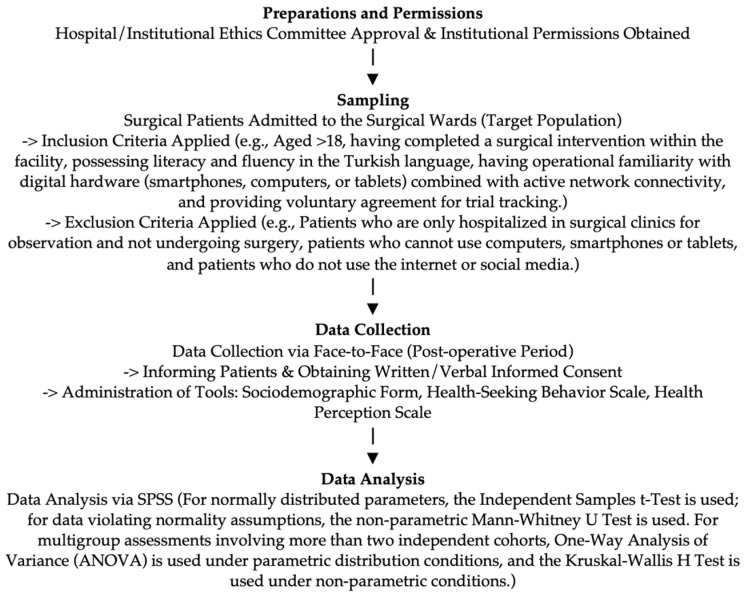
Flowchart of the Study Procedure.

The detailed step-by-step procedure followed during this investigation, including sample selection, ethical considerations, and data collection phases, is systematically illustrated in Figure 1.

### 2.5. Data Analysis

Statistical evaluations of the collected research data were executed utilizing IBM SPSS Statistics, Version 27.0 (IBM Corp., Armonk, NY, USA). The baseline characteristics of the variables were documented through descriptive statistics, including frequencies, percentages, means, medians, standard deviations, as well as minimum and maximum boundaries. To evaluate whether the data conformed to a parametric distribution, skewness and kurtosis indices were thoroughly scrutinized. When exploring statistical variations across two autonomous categories, the Independent Sample t-Test was deployed for normally distributed parameters, whereas the non-parametric Mann–Whitney U Test was utilized for data violating normality assumptions. For multi-group evaluations encompassing more than two independent cohorts, One-Way Analysis of Variance (ANOVA) was preferred under parametric distribution conditions, while the Kruskal–Wallis H Test was administered under non-parametric circumstances. In cases where significant statistical fluctuations were documented across multiple independent groups for continuous metrics, post hoc paired tracking tests were initiated to distinguish the exact groups introducing the variance. Inter-variable dynamics among continuous parameters were measured using Pearson’s correlation matrices, with all analytical computations based on a 95% confidence interval framework (*p* = 0.05).

### 2.6. Ethical Considerations

Institutional ethics clearance for the execution of this investigation was granted by the Medical Research Ethics Committee of Ege University, the facility where the trial was carried out (protocol certificate registration: 23-10.1T/36, issued on 20 October 2022). Formal utilization privileges for the respective data tracking scales were successfully acquired from their original developers via digital correspondence. All research protocols involving human subjects were strictly maintained and performed in complete alignment with the ethical regulations of the responsible local or national oversight boards, the mandates of the 1964 Declaration of Helsinki along with its later revisions, or equivalent standardized ethical principles.

## 3. Results

The mean age of the studied patient cohort was found to be 53.08 ± 15.80 years. Within the sample distribution, male participants accounted for 50.5% and 34.6% of the overall cohort possessed a elementary school education level (Table 1).

In terms of digital connectivity infrastructure, a vast majority of the patients (94.7%) accessed the internet primarily via their mobile phones. Regarding daily connectivity duration, 41.2% of the participants utilized the internet for ≤1 h per day. Social media platform preferences revealed that Facebook and Instagram were utilized by 50.5% and 50.8% of the cohort, respectively. Furthermore, 62.2% of the individuals reported a history of searching the internet for general health-related information, 60.9% specifically searched for data regarding their current clinical condition, and 58.5% expressed partial trust in the medical information obtained online (Table 2).

The descriptive analysis of the measurement instruments showed that the participants’ overall mean score on the Health Search Behavior Scale was 38.13 ± 7.02 (min: 21, max: 56). Concurrently, the cumulative mean score for the Health Perception Scale was determined to be 40.90 ± 4.25 (min: 29, max: 57). To confirm internal consistency, Cronbach’s alpha reliability coefficients were calculated, yielding values of 0.75 for the Health Search Behavior Scale and 0.77 for the Health Perception Scale (Table 3).

The statistical tracking models revealed no significant correlation between the cumulative outcomes of the Health-Seeking Behavior Scale and the overall Perception of Health Scale (r = −0.044; *p* = 0.398). However, specific sub-dimension interactions demonstrated noteworthy dynamics. Digital medical information tracking (“online health-seeking behavior”) demonstrated a weak positive linear correlation with the Control Center subscale (r = 0.255; *p* < 0.001). In contrast, this digital tracking parameter exhibited very weak negative associations with both the Importance of Health (r = −0.171) and Self-Awareness (r = −0.136) metrics (*p* < 0.05) while maintaining a very weak positive linear relationship with the cumulative Health Perception summary metric (r = 0.105; *p* < 0.05). Examination of clinical consultation pathways (“Professional Health-Seeking Behavior”) reflected a very weak positive link with Self-Awareness (r = 0.198; *p* < 0.001) but displayed a weak negative interactive dynamic with Certainty (r = −0.291; *p* < 0.001). This professional healthcare utilization domain also established very weak negative correlations with the Control Center (r = −0.136) and the aggregate Health Perception summary index (r = −0.175) (*p* < 0.05). Finally, a negligible yet positive linear correlation was identified between overall health-seeking inclinations (“total Health-Seeking Behavior”) and the Locus of Control sub-dimension (r = 0.153; *p* < 0.05) (Table 4).

Comparative inferential statistics revealed that participants younger than 54 years old exhibited a significantly higher online health search score (15.79 ± 5.50) compared to those aged 54 and above (14.10 ± 5.83), demonstrating a statistically significant variation between the two age brackets (t = 2.886; *p* < 0.05). When analyzed by gender, female participants displayed significantly higher scores in both online health search and total health search metrics compared to males (t = 2.316; *p* < 0.05 and t = 2.130; *p* < 0.05). Educational stratification also yielded significant variations across online health searches (F = 7.690; *p* < 0.001), total health searches (F = 5.655; *p* < 0.001), locus of control (F = 9.877; *p* < 0.001), and the importance of health (F = 7.080; *p* < 0.001). Post hoc analyses confirmed that elementary school graduates scored significantly lower than all other educational groups. Furthermore, variations based on internet usage duration were significant for online health searches (F = 4.791; *p* < 0.05), total health searches (F = 4.232; *p* < 0.05), and health information centers (F = 3.549; *p* < 0.05) with individuals using the internet for ≤1 h per day demonstrating lower scores. Finally, individual social media platform preferences heavily influenced scale outcomes. Instagram users exhibited significantly higher scores for online health searches (t = 6.579; *p* < 0.001), total health searches (t = 6.002; *p* < 0.001), control center dimensions (t = 3.894; *p* < 0.001), and the importance of health (t = −2.369; *p* < 0.05). Twitter users displayed higher online health search behaviors (t = 2.427; *p* < 0.05), while WhatsApp users similarly reflected higher scores for both online (t = 3.619; *p* < 0.001) and total health searches (t = 3.199; *p* < 0.05). Conversely, non-users of these respective platforms demonstrated significantly lower baseline scores across online health searches (t = −5.313; *p* < 0.001), total health searches (t = −4.607; *p* < 0.001), locus of control (t = −1.974; *p* < 0.05), and the importance of health scales (t = 2.863; *p* < 0.05) (Table 5).

## 4. Discussion

In this study, the mean score on the Patients’ Health-Seeking Behavior Scale was 38.13 ± 7.02, while the mean score on the Health Perception Scale was 40.90 ± 4.25. These findings indicate that patients’ health-seeking behaviors are at a moderate level, while their health perceptions are at a positive level. Recent studies indicate that health-seeking behavior is influenced by multidimensional factors such as health literacy, access to digital resources, and patient engagement [21]. The outcomes derived from this research exhibit strong alignment with existing scholarly literature, as enhanced scores in health perception indicate that individuals maintain a positive self-evaluation regarding their general medical status, which, in turn, serves as a constructive catalyst in reinforcing healthy behavioral practices [22]. Notably, our statistical analysis revealed no significant correlation between the cumulative total scores of the Health-Seeking Behavior Scale and the overall Perception of Health Scale. Although the literature generally reports positive relationships between these two variables [23], this prominent null finding constitutes a major conceptual outcome of our investigation. This lack of significant global correlation carries important theoretical and practical implications for perioperative care. Theoretically, it suggests that a patient’s cognitive perception of their health does not automatically translate into behavioral actions regarding online or offline health-seeking information. In a surgical inpatient context, health-seeking behaviors may be driven more heavily by immediate situational factors—such as acute preoperative anxiety, specific procedural uncertainties, or fear of surgical outcomes—rather than the patient’s baseline, steady-state health perception. Furthermore, this discrepancy highlights that digital information-seeking in modern healthcare is a highly fragmented phenomenon. Patients with a positive health perception may still engage in intensive online searches due to curiosity or reassurance-seeking, while those with poor health perceptions might experience information avoidance due to digital fatigue or fear of self-diagnosis. Consequently, evaluating these two constructs purely through aggregate total scores overshadows the complex, multidimensional nature of patient behavior, as evidenced by the distinct, divergent correlations observed among specific subscales. In this context, age emerges as a pivotal social determinant governing online health information-seeking behavior. Younger cohorts within the surgical patient population tend to navigate social media platforms with greater agility, translating into higher digital health literacy and more proactive health-seeking actions. Conversely, older adults frequently encounter structural and cognitive barriers in deciphering online medical data, a phenomenon heavily documented in recent international literature as the ‘digital health divide’ [24]. From a public health perspective, this age-related disparity emphasizes the need for age-tailored digital navigation support in surgical wards to ensure equitable access to perioperative information.

The documentation of a weak yet positive linear relationship linking web-based medical information tracking with dimensions of perceived personal control suggests that an escalating sense of autonomy over one’s physiological well-being actively stimulates an individual’s inclination to conduct digital data investigations [25]. In contrast, the identification of very weak negative correlations with the importance of health and self-awareness suggests that, despite valuing their health, individuals do not always exhibit active information-seeking behavior [26]. Furthermore, the significant variations observed across gender subgroups underscore the distinct socio-cultural roles individuals assume regarding health responsibilities. Recent studies globally indicate that women often display higher engagement in online health-seeking behaviors, driven by a higher baseline of health awareness and their frequent role as primary health decision-makers within family structures [27]. However, this gendered pattern is intricately linked with broader social determinants, including educational background and access to verified digital infrastructure, which can modify how surgical patients perceive digital health risks and select information channels [28]. The finding of a very weak positive relationship between online health-seeking behavior and health perception aligns with the limited relationships between health information-seeking behavior and health evaluations noted in the literature [22]. The finding of a very weak positive association between professional healthcare-seeking behavior and self-awareness in this study suggests a positive relationship where individuals who know themselves better also report a higher tendency to seek professional healthcare services [29]. In contrast, the identification of negative correlations with certainty and locus of control suggests that individuals with a high perceived sense of control over their health may have a reduced need to seek professional help [30,31]. The finding of a very weak negative relationship between professional healthcare-seeking behavior and health perception suggests that individuals may be less likely to seek healthcare services when they perceive their health to be good. Additionally, the positive relationship between the total score of healthcare-seeking behavior and the locus of control supports the notion that health behaviors are associated with the perception of control [32].

The prominent level of web-based information search actions observed among participants below 54 years of age within our cohort can be explained by the technological competence and daily digital engagement characteristic of younger demographics [33]. Prior literature supports this correlation, highlighting a historical negative relationship between chronological age and electronic health literacy, whereby youthful cohorts navigate virtual networks with greater agility to access medical data [34].

The documented disparity revealing superior mean scores among female patients across both web-based and general monitoring categories suggests a heightened health vigilance and a proactive attitude toward clinical engagement among women. This trend closely matches global data, which frequently validates that female cohorts engage in intensive medical investigations over virtual networks more regularly than men [2,35]. The increase in online health-seeking behavior and health-seeking behavior scores as educational level rises is directly related to health literacy. It is known that individuals with higher educational levels possess more developed skills in both accessing and evaluating information. According to the conceptual framework established by the World Health Organization, health literacy represents a person’s cognitive and social ability to acquire, comprehend, and implement information pertaining to health conditions; furthermore, the institution underscores that this proficiency is intrinsically connected to an individual’s academic background [36].

The higher levels of online health-seeking behavior observed among individuals with extended time spent on the internet coincide with individuals’ growing exposure to digital environments. As daily internet usage increases, so does the likelihood that individuals will encounter health-related content and explore it further. This trend indicates that digital platforms are increasingly being used as sources of health information [35].

The positive relationship between social media use and online health-seeking behavior suggests that social media platforms have become important sources of health information. Recent studies indicate that social media is significantly associated with individuals’ health behaviors, awareness, and decision-making processes [37]. Platforms such as Instagram, Twitter, and WhatsApp facilitate the rapid sharing of health information and user experiences, thereby supporting information exchange and awareness. Messaging applications like WhatsApp enable fast dissemination of health-related content through group communication. In contrast, lower levels of health information-seeking observed among users of other platforms may be related to their primarily entertainment-oriented content and limited health-related information.

### 4.1. Study Limitations

The use of a cross-sectional design in this study limits the ability to establish definitive causal relationships between social media use, health perceptions, and health-seeking behaviors. This study was conducted at a single center; this also limits the generalizability of the findings to larger patient populations or different clinical contexts.

### 4.2. Practical Implications

Despite these limitations, the findings offer vital implications for clinical practice, patient education, and the development of digital health interventions for surgical patients. Healthcare providers, particularly surgical nurses, should assess patients’ digital health literacy in the preoperative period. For patients who actively use social media to shape their perceptions of health, clinicians can develop evidence-based digital resources and direct patients to validated online platforms. This approach can minimize preoperative anxiety and improve patient engagement and postoperative recovery outcomes by eliminating misinformation.

### 4.3. Future Research Directions

Future research should utilize longitudinal designs or multi-center methodologies to track how surgical patients’ health-seeking behaviors evolve throughout the entire perioperative trajectory. Furthermore, qualitative or mixed-method studies are warranted to explore the underlying causal mechanisms driving digital health literacy and social media usage. Investigating the direct impact of specific digital health education interventions on surgical patient outcomes remains an essential area for subsequent scholarly inquiry.

## 5. Conclusions

The outcomes of this investigation demonstrate that surgical patients’ digital health information acquisition tendencies and health display significant variations across distinct sociodemographic characteristics and digital platform usage patterns. Younger age, female gender, and higher educational attainment stand out as key drivers of intensive engagement in online health-seeking behaviors. Furthermore, increased daily internet exposure to and active participation on mainstream social media platforms—particularly Instagram, Twitter, and WhatsApp—are closely linked with higher rates of virtual medical information tracking.

Based on these findings, the results highlight the need for targeted approaches in clinical settings. Instead of a general strategy, digital health literacy initiatives should be tailored to bridge the gaps among older populations and individuals with limited educational profiles. Healthcare providers, particularly surgical nursing specialists, should actively integrate digital health referrals into preoperative patient education. Implementing strategic interventions that guide surgical patients to validated, evidence-based digital resources remain a fundamental public health necessity to maximize the benefits of online resources while minimizing the risks of digital misinformation throughout the surgical process.

## Figures and Tables

**Table 1:** Demographic Characteristics of Patients.

	X ± SD	M (Min–Max)
**Age**	53.08 ± 15.80	54.0 (18–86)
	***n***	**%**
**Gender**		
Female	186	49.5
Male	190	50.5
**Education Level**		
Elementary School	130	34.6
Middle School	49	13.0
High School	100	26.6
Associate’s degree	22	5.9
Bachelor’s Degree	67	17.8
Graduate Degree	7	1.9
**Total**	**376**	**100**

**Table 2:** Patients’ Characteristics Regarding Internet and Social Media Usage Patterns.

	*n*	%
**Device Used to Access the Internet** **		
Phone	356	94.7
Tablet	34	9.0
Computer	44	11.7
Other	14	3.7
**Time Spent Online**		
1 h or Less	155	41.2
2–3 h	149	39.6
4–5 h	46	12.2
6 h or More	26	6.9
**Social Media Platform Used**		
Facebook	190	50.5
Instagram	191	50.8
Twitter	70	18.6
WhatsApp	243	64.6
Other	74	19.7
**Previous Health-Related Online Searches**		
Yes	234	62.2
No	142	37.8
**Current Health-Related Online Searches**		
Yes	229	60.9
No	147	39.1
**Trust in Online Health Information**		
Yes	70	18.6
No	86	22.9
Partially	220	58.5
**Total**	**376**	**100**

** Participants could select more than one option.

**Table 3:** Patients’ Scores on the Health-Seeking Behavior Scale and the Health Perception Scale.

	X ± SD	M (Min–Max)
**Health-Seeking Behavior Scale**		
Online Health-Seeking Behavior	14.93 ± 5.73	14.5 (6–30)
Professional Health-Seeking Behavior	13.86 ± 1.70	15.0 (6–15)
Traditional Health-Seeking Behavior	9.35 ± 3.07	10.0 (3–15)
Health-Seeking Behavior Scale—Total	38.13 ± 7.02	38.0 (21–56)
**Health Perception Scale**		
Control Center	13.78 ± 4.16	14.0 (5–23)
Certainty	8.23 ± 2.71	8.0 (4–18)
Importance of Health	9.14 ± 2.73	9.0 (3–15)
Self-Awareness	9.75 ± 2.35	10.0 (3–15)
Health Perception Scale—Total	40.90 ± 4.25	41.0 (29–57)

**Table 4:** The Relationship Between the Health-Seeking Behavior Scale and the Health Perception Scale Among Patients.

Health Perception Scale	Health-Seeking Behavior Scale							
	Online Health-Seeking Behavior		Professional Health-Seeking Behavior		Traditional Health-Seeking Behavior		Health-Seeking Behavior Scale—Total	
	r	*p*	r	*p*	r	*p*	r	*p*
Control Center	0.255	<0.001 **	−0.136	0.008 *	−0.051	0.325	0.153	0.003 *
Certainty	0.064	0.217	−0.291	<0.001 **	−0.074	0.152	−0.051	0.327
Importance of Health	−0.171	0.001 *	0.053	0.305	0.070	0.173	−0.096	0.064
Self-Awareness	−0.136	0.008 *	0.198	<0.001 **	0.096	0.063	−0.021	0.681
Health Perception Scale—Total	0.105	0.043 *	−0.175	0.001 *	0.002	0.975	0.044	0.398

Pearson’s correlation, * *p* < 0.05, ** *p* < 0.001, Correlated factors are indicated in bold.

**Table 5:** Patients’ Scores on the Health-Seeking Behavior Scale and the Health Perception Scale by Variable.

Characteristics	Health-Seeking Behavior Scale				Health Perception Scale				
	Online Health-Seeking Behavior X ± SD	Professional Health-Seeking Behavior X ± SD	Traditional Health-Seeking Behavior X ± SD	Health-Seeking Behavior Scale—Total X ± SD	Control Center X ± SD	Certainty X ± SD	Importance of Health X ± SD	Self-Awareness X ± SD	Health Perception Scale—Total X ± SD
**Age** Under 54 54 and older	15.79 ± 5.50 14.10 ± 5.83 t ^a^/Z ^b^: 2.886 ^a^ ***p*: 0.004**	13.84 ± 1.70 13.88 ± 1.71 t ^a^/Z ^b^: −0.555 ^b^ *p*: 0.579	9.21 ± 3.02 9.48 ± 3.12 t ^a^/Z ^b^: −0.865 ^a^ *p*: 0.387	38.84 ± 6.93 37.47 ± 7.05 t ^a^/Z ^b^: 1.898 ^a^ *p*: 0.058	14.04 ± 4.15 13.54 ± 4.17 t ^a^/Z ^b^: 1.152 ^a^ *p*: 0.250	8.33 ± 2.68 8.13 ± 2.73 t ^a^/Z ^b^: 0.709 ^a^ *p*: 0.479	8.95 ± 2.75 9.32 ± 2.71 t ^a^/Z ^b^: −1.334 ^a^ *p*: 0.183	9.76 ± 2.54 9.75 ± 2.17 t ^a^/Z ^b^: 0.055 ^a^ *p*: 0.956	41.07 ± 4.19 40.74 ± 4.32 t ^a^/Z ^b^: 0.752 ^a^ *p*: 0.453
**Gender** Female Male	15.61 ± 5.71 14.25 ± 5.68 t ^a^/Z ^b^: 2.316 ^a^ ***p*: 0.021**	13.92 ± 1.79 13.80 ± 1.61 t ^a^/Z ^b^: −1.315 ^b^ *p*: 0.189	9.38 ± 3.06 9.32 ± 3.09 t ^a^/Z ^b^: 0.174 ^a^ *p*: 0.862	38.91 ± 7.02 37.37 ± 6.95 t ^a^/Z ^b^: 2.130 ^a^ ***p*: 0.034**	13.87 ± 4.01 13.70 ± 4.31 t ^a^/Z ^b^: 0.398 ^a^ *p*: 0.691	8.22 ± 2.48 8.23 ± 2.92 t ^a^/Z ^b^: −0.040 ^a^ *p*: 0.968	8.94 ± 2.68 9.34 ± 2.78 t ^a^/Z ^b^: −1.425 ^a^ *p*: 0.155	9.62 ± 2.29 9.88 ± 2.42 t ^a^/Z ^b^: −1.052 ^a^ *p*: 0.294	40.65 ± 3.84 41.15 ± 4.62 t ^a^/Z ^b^: −1.133 ^a^ *p*: 0.258
**Education Level** Elementary School (1) Middle School (2) High School (3) Associate’s degree (4) Bachelor’s Degree (5) Graduate degree (6)	12.85 ± 5.38 13.98 ± 5.24 16.23 ± 5.32 16.91 ± 5.98 16.73 ± 5.99 18.29 ± 5.19 F ^c^/KW ^d^:7.690 ^c^ ***p* < 0.001** Difference ^e^: 1 < 3, 1 < 4, 1 < 5, 1 < 6	13.88 ± 1.65 13.71 ± 1.70 13.87 ± 1.72 13.59 ± 2.15 13.99 ± 1.67 13.71 ± 1.38 F ^c^/KW ^d^: 2.036 ^d^ *p*: 0.844	9.24 ± 3.48 9.08 ± 3.05 9.75 ± 2.64 9.05 ± 2.84 9.22 ± 2.88 9.00 ± 3.46 F ^c^/KW ^d^: 0.530 ^c^ *p*: 0.754	35.97 ± 7.16 36.78 ± 6.07 39.85 ± 6.21 39.55 ± 8.37 39.94 ± 6.99 41.00 ± 6.51 F ^c^/KW ^d^: 5.655 ^c^ ***p*: < 0.001** Difference ^e^: 1 < 3, 1 < 5	12.05 ± 4.04 13.84 ± 3.60 14.15 ± 4.12 15.73 ± 3.73 15.79 ± 3.76 15.00 ± 3.87 F ^c^/KW ^d^: 9.877 ^c^ ***p* < 0.001** Difference ^e^: 1 < 3, 1 < 4, 1 < 5	8.24 ± 2.89 7.71 ± 2.56 8.39 ± 2.81 8.14 ± 2.47 8.27 ± 2.47 8.57 ± 1.51 F ^c^/KW ^d^: 0.452 ^c^ *p*: 0.812	10.14 ± 2.59 9.0 ± 2.69 8.79 ± 2.72 8.23 ± 2.39 8.03 ± 2.63 8.71 ± 2.06 F ^c^/KW ^d^: 7.080 ^c^ ***p* < 0.001** Difference ^e^: 1 < 3, 1 < 4, 1 < 5	10.22 ± 2.37 9.45 ± 2.0 9.68 ± 2.53 9.09 ± 2.27 9.43 ± 2.16 8.86 ± 1.07 F ^c^/KW ^d^: 2.050 ^c^ *p*: 0.071	40.65 ± 3.93 40.10 ± 4.22 41.01 ± 4.70 41.18 ± 4.83 41.52 ± 3.91 41.14 ± 3.13 F ^c^/KW ^d^: 0.763 ^c^ *p*: 0.577
**Time Spent Online** 1 h or Less (1) 2–3 h (2) 4–5 h (3) 6 h or More (4)	13.63 ± 5.75 15.72 ± 5.48 15.76 ± 5.47 16.58 ± 6.17 F ^c^/KW ^d^: 4.791 ^c^ ***p* < 0.003** Difference ^e^: 1 < 2, 1 < 3, 1 < 4	13.74 ± 1.80 13.82 ± 1.67 14.24 ± 1.18 14.12 ± 1.97 F ^c^/KW ^d^: 4.186 ^d^ *p* < 0.242	9.34 ± 3.17 9.18 ± 2.99 9.96 ± 2.84 9.31 ± 3.36 F ^c^/KW ^d^: 0.750 ^c^ *p* < 0.523	36.71 ± 6.97 38.72 ± 6.93 39.96 ± 6.69 40.00 ± 7.09 F ^c^/KW ^d^: 4.232 ^c^ ***p* < 0.006** Difference ^e^: 1 < 3, 1 < 4	13.11 ± 4.28 14.07 ± 4.23 15.24 ± 3.45 13.62 ± 3.50 F ^c^/KW ^d^: 3.549 ^c^ ***p* < 0.015** Difference ^e^: 1 < 3	8.40 ± 3.01 8.15 ± 2.27 8.00 ± 2.84 8.00 ± 2.98 F ^c^/KW ^d^: 0.413 ^c^ *p* < 0.744	9.48 ± 2.82 9.03 ± 2.70 8.48 ± 2.61 8.88 ± 2.45 F ^c^/KW ^d^: 1.890 ^c^ *p* < 0.131	9.95 ± 2.40 9.60 ± 2.34 9.33 ± 1.96 10.19 ± 2.68 F ^c^/KW ^d^: 1.365 ^c^ *p* < 0.253	40.94 ± 4.21 40.85 ± 4.09 41.04 ± 4.24 40.69 ± 5.53 F ^c^/KW ^d^: 0.049 ^c^ *p* < 0.986
**Social Media Platform Used—Facebook** Yes No	15.47 ± 5.61 14.37 ± 5.82 t ^a^/Z ^b^: 1.874 ^a^ *p*: 0.062	13.86 ± 1.67 13.85 ± 1.73 t ^a^/Z ^b^: −0.086 ^b^ *p*: 0.932	9.42 ± 2.95 9.31 ± 3.17 t ^a^/Z ^b^: 0.324 ^a^ *p*: 0.746	38.75 ± 7.10 37.53 ± 6.90 t ^a^/Z ^b^: 1.690 ^a^ *p*: 0.092	13.93 ± 4.18 13.62 ± 4.15 t ^a^/Z ^b^: 0.720 ^a^ *p*: 0.472	8.18 ± 2.43 8.30 ± 2.96 t ^a^/Z ^b^: −0.423 ^a^ *p*: 0.672	9.13 ± 2.70 9.17 ± 2.77 t ^a^/Z ^b^: −0.146 ^a^ *p*: 0.884	9.69 ± 2.37 9.82 ± 2.35 t ^a^/Z ^b^: −0.521 ^a^ *p*: 0.603	40.93 ± 4.23 40.91 ± 4.27 t ^a^/Z ^b^: 0.053 ^a^ *p*: 0.957
**Social Media Platform Used—Instagram** Yes No	16.74 ± 5.55 13.05 ± 5.30 t ^a^/Z ^b^: 6.579 ^a^ ***p* < 0.001**	13.91 ± 1.59 13.81 ± 1.81 t ^a^/Z ^b^: −0.101 ^b^ *p*: 0.920	9.53 ± 2.91 9.16 ± 3.22 t ^a^/Z ^b^: 1.192 ^a^ *p*: 0.234	40.18 ± 6.69 36.02 ± 6.74 t ^a^/Z ^b^: 6.002 ^a^ ***p* < 0.001**	14.59 ± 3.93 12.95 ± 4.24 t ^a^/Z ^b^: 3.894 ^a^ ***p* < 0.001**	8.15 ± 2.57 8.30 ± 2.85 t ^a^/Z ^b^: −0.540 ^a^ *p*: 0.590	8.81 ± 2.72 9.48 ± 2.71 t ^a^/Z ^b^: −2.369 ^a^ ***p*: 0.018**	9.63 ± 2.40 9.88 ± 2.31 t ^a^/Z ^b^: −0.997 ^a^ *p*: 0.319	41.19 ± 4.38 40.61 ± 4.11 t ^a^/Z ^b^: 1.330 ^a^ *p*: 0.184
**Social Media Platform Used—Twitter** Yes No	16.41 ± 5.49 14.58 ± 5.73 t ^a^/Z ^b^: 2.427 ^a^ ***p*: 0.016**	13.80 ± 1.91 13.87 ± 1.65 t ^a^/Z ^b^: −0.158 ^b^ *p*: 0.875	8.74 ± 2.94 9.49 ± 3.09 t ^a^/Z ^b^: −1.835 ^a^ *p*: 0.067	38.96 ± 6.95 37.94 ± 7.03 t ^a^/Z ^b^: 1.089 ^a^ *p*: 0.277	13.71 ± 4.06 13.80 ± 4.19 t ^a^/Z ^b^: −0.156 ^a^ *p*: 0.876	8.17 ± 2.80 8.24 ± 2.69 t ^a^/Z ^b^: −0.187 ^a^ *p*: 0.852	8.80 ± 2.74 9.22 ± 2.73 t ^a^/Z ^b^: −1.148 ^a^ *p*: 0.252	9.59 ± 2.37 9.79 ± 2.35 t ^a^/Z ^b^: −0.657 ^a^ *p*: 0.511	40.27 ± 4.58 41.05 ± 0.17 t ^a^/Z ^b^: −1.376 ^a^ *p*: 0.170
**Social Media Platform Used—WhatsApp** Yes No	15.70 ± 5.74 13.50 ± 5.44 t ^a^/Z ^b^: 3.619 ^a^ ***p* < 0.001**	13.95 ± 1.60 13.70 ± 1.86 t ^a^/Z ^b^: −0.900 ^b^ *p*: 0.368	9.33 ± 3.02 9.38 ± 3.17 t ^a^/Z ^b^: −0.164 ^a^ *p*: 0.870	38.98 ± 7.17 36.59 ± 6.49 t ^a^/Z ^b^: 3.199 ^a^ ***p*: 0.001**	14.08 ± 4.06 13.24 ± 4.31 t ^a^/Z ^b^: 1.882 ^a^ *p*: 0.061	8.27 ± 2.63 8.14 ± 2.85 t ^a^/Z ^b^: 0.440 ^a^ *p*: 0.660	8.97 ± 2.68 9.44 ± 2.81 t ^a^/Z ^b^: −1.605 ^a^ *p*: 0.109	9.70 ± 2.42 9.86 ± 2.24 t ^a^/Z ^b^: −0.636 ^a^ *p*: 0.525	41.02 ± 4.14 40.68 ± 4.46 t ^a^/Z ^b^: 0.733 ^a^ *p*: 0.464
**Social Media Platforms Used—Other (e.g., TikTok, Snapchat, YouTube)** Yes No	11.86 ± 5.26 15.68 ± 5.59 t ^a^/Z ^b^: −5.313 ^a^ ***p* < 0.001**	13.82 ± 1.55 13.87 ± 1.74 t ^a^/Z ^b^: −0.671 ^b^ *p*: 0.502	9.16 ± 3.22 9.39 ± 3.04 t ^a^/Z ^b^: −0.582 ^a^ *p*: 0.561	34.85 ± 5.97 38.94 ± 7.03 t ^a^/Z ^b^: −4.607 ^a^ ***p* < 0.001**	12.93 ± 4.60 13.99 ± 4.03 t ^a^/Z ^b^: −1.974 ^a^ ***p*: 0.049**	8.09 ± 3.02 8.26 ± 2.63 t ^a^/Z ^b^: −0.466 ^a^ *p*: 0.642	9.95 ± 2.74 8.94 ± 2.70 t ^a^/Z ^b^: 2.863 ^a^ ***p*: 0.004**	9.74 ± 2.39 9.75 ± 2.35 t ^a^/Z ^b^: −0.038 ^a^ *p*: 0.969	40.72 ± 4.32 40.95 ± 4.24 t ^a^/Z ^b^: −0.418 *p*: 0.676

^a^: Independent Sample *t*-Test, Z ^b^: Mann–Whitney U Test, ^c^: One-Way Analysis of Variance, ^d^: Kruskal–Wallis H Test, ^e^: Bonferroni Post Hoc Test, Bold values indicate statistically significant differences (*p* < 0.05).

## Data Availability

Data are contained within this article. The original contributions presented in this study are included in this article. Further inquiries can be directed to the corresponding author.
